# The Epidemiological Boehringer Ingelheim Employee Study (Part 3): Association of Elevated Fasting Insulin Levels but Not HOMA-IR With Increased Intima Media Thickness and Arteriosclerosis in Middle-Aged Persons

**DOI:** 10.3389/fcvm.2021.752789

**Published:** 2021-10-22

**Authors:** Martin Röhling, Kerstin Kempf, Hubert Kolb, Tobias Martin, Michael Schneider, Stephan Martin

**Affiliations:** ^1^West-German Center of Diabetes and Health, Düsseldorf Catholic Hospital Group, Düsseldorf, Germany; ^2^Faculty of Medicine, Heinrich Heine University Düsseldorf, Düsseldorf, Germany; ^3^Occupational Health and Medical Services, Boehringer Ingelheim Pharma GmbH & Co. KG, Ingelheim am Rhein, Germany

**Keywords:** obesity, hyperinsulinemia/insulin resistance, cardiovascular disease, arteriosclerosis, arterial lesions

## Abstract

**Background:** Recently published genetic studies have indicated a causal link between elevated insulin levels and cardiovascular disease (CVD) risk. We, therefore, hypothesized that increased fasting insulin levels are also associated with precursors of CVD such as endothelial lesions.

**Methods:** Middle-aged (≥40 years, *n* = 1,639) employees were followed up for the occurrence of increased intima media thickness (IMT ≥ 1 mm) or plaques in abdominal or cervical arteries (arteriosclerosis). Multivariable logistic regression analyses determined the incidence of increased IMT or arteriosclerosis. Adjusted relative risk (ARR) for increased IMT and arteriosclerosis was calculated by using Mantel-Haenszel analysis.

**Results:** Increased IMT was diagnosed in 238 participants (15 %) and 328 (20 %) developed arteriosclerosis after 5 years of follow-up. Logistic regression analysis identified fasting insulin, BMI and smoking as risk factors for both cardiovascular endpoints (all *p* < 0.05), whereas age and diastolic blood pressure were risk factors for increased IMT only, and male sex was associated with incident arteriosclerosis only (all *p* < 0.01). Additional adjustment for BMI change during follow-up did not modify these associations (including fasting insulin), but adjustment for fasting insulin change during follow-up removed BMI as risk factor for both cardiovascular endpoints. Fasting insulin change during follow-up but not BMI change associated with increased IMT and arteriosclerosis (both *p* < 0.001). ARR analysis indicated that high fasting insulin and BMI added to age and sex as risk factors. Homeostatic model assessment of insulin resistance (HOMA-IR) did not associate with either cardiovascular endpoint in any model and smoking did not increase the risk conferred by high fasting insulin levels.

**Conclusions:** Higher fasting insulin levels and increases in fasting insulin over time are associated with atherogenic progression and supersede BMI as well as HOMA-IR as risk factors.

## Introduction

Cardiovascular diseases (CVD) are the leading cause for overall mortality worldwide (1). One major contributing factor, besides smoking, age, sex, and blood pressure, in this context is overweight or obesity, respectively (2). To date, numerous studies have reported positive associations between overweight or central fat distribution and risk of fatal or non-fatal cardiovascular events (CVE) (3) as well as atherogenic progression (4). It has been suggested that hyperinsulinemia, which promotes the development of obesity by inhibiting lipolysis (5) and inducing lipogenesis (6), is a further major independent contributing factor for the development of CVD's and CVE's (7–9). In this context, recently published genetic studies indicated for the first time a causal link between elevated insulin levels and CVD risk (10, 11). Furthermore, several mechanistic (12) and observational studies (13) have shown positive associations between elevated insulin levels and an increase in cardiovascular and all-cause mortality in different populations. Particularly, the results of mechanistic studies indicate an atherogenic effect of persistently high insulin levels, probably via endothelial dysfunction (14), a pro-inflammatory activity of macrophages (15), suppression of autophagy and an increase in protein synthesis (16), as well as a compromised cytoprotective response to oxidative and other chemical stress (17).

So far, primarily cross-sectional studies (18–21) investigated the interrelation of insulin levels with the occurrence of CVD and CVE (22, 23) or atherogenic progression (9, 19, 24), respectively. Furthermore, prospective studies are rather rare (25), and the generalizability of findings of the examined populations is somewhat reduced [e.g., Pima Indians (26), older-aged persons with higher fasting insulin levels (27)]. Moreover, existing publications in this context are characterized by only small cohorts (28–32), and the major part of these studies focusses on insulin resistance (e.g., homeostatic model assessment of insulin resistance (HOMA-IR) index) and/or glucose levels (33–37) as potential glucometabolic predictors, and insulin levels in blood are only of inferior priority. However, the relevance of HOMA-IR as measure of total body, peripheral or hepatic insulin resistance has been questioned (38).

Therefore, more longitudinal studies are needed to investigate the impact of insulin levels compared with HOMA-IR on atherogenic progression. In the present longitudinal study, we have investigated the incidence of increased intima media thickness (IMT) and arteriosclerosis as precursors of atherogenic progression in a prospective occupational health care setting in middle-aged employees. This allowed us to evaluate metabolic and other parameters as risk factors of arterial lesions.

## Materials and Methods

### Study Design and Population

Details of the open-label Boehringer Ingelheim (BI) employee cohort have been described previously (39). In short, BI employees aged ≥40 and employed for at least 2 years were offered to participate in a comprehensive health check program of the BI corporate medical department every 3–5 years free of charge. As part of an occupational health care initiative this program is still ongoing. For the present analysis data from those participants who joined the program between 01.01.2006 and 31.12.2017 were analyzed. The study was approved by the ethics committee of the Ärztekammer Nordrhein (no. 2011340). All clinical investigations have been conducted according to the principles of the Declaration of Helsinki and participants gave written informed consent.

### Outcomes

Anthropometrical [body-mass-index (BMI), weight], clinical (systolic and diastolic blood pressure), laboratory [triglycerides, total cholesterol, high-density lipoprotein cholesterol (HDL-C), low-density lipoprotein cholesterol (LDL-C), hemoglobin A1c (HbA1c), fasting blood glucose, fasting insulin, homeostasis model assessment of insulin resistance (HOMA-IR)], and behavioral (smoking) parameters were determined as previously reported (39).

Cardiometabolic diseases were defined as previously described [hypercholesterolemia (≥200 mg/dl), hypertension (systolic blood pressure: ≥140 mmHg and/or diastolic blood pressure: ≥90 mmHg or self-reported), hypertriglyceridemia (triglycerides: ≥150 mg/dl), insulin resistance (HOMA-IR: ≥2.6), hyperinsulinemia (fasting insulin: >15 μU/ml) and type 2 diabetes mellitus (fasting blood glucose: ≥126 mg/dl and/or HbA1c ≥ 6.5%)] (39).

Arteriosclerosis [occurrence of plaques in abdominal arteries (aorta abdominalis) and/or neck arteries (aorta carotis) and/or stenoses in neck arteries (aorta carotis)] and increased IMT (>1 mm) were determined as early markers of cardiovascular disease.

### Statistical Analysis

Shown are means with standard deviation (SD) [mean (SD)] for normally distributed variables or median and interquartile ranges for variables with skewed distribution. Dichotomous variables were analyzed by Chi-square test or McNemar test, as appropriate.

Multivariable logistic regression analyses were performed to investigate the influence of anthropometric, clinical, and laboratory parameters on the incidence of increased IMT or arteriosclerosis expressed as odds ratio (OR) with 95% confidence interval (CI). Logistic regression analyses were adjusted for the baseline values age, sex, smoking, BMI, fasting insulin, fasting blood glucose, systolic blood pressure, diastolic blood pressure, HDL-C, LDL-C, total cholesterol, triglycerides, HbA1c, HOMA-IR (Model 1) or “BMI change” during follow-up (Model 2= Model 1 + “BMI change”) or “insulin change” during follow-up (Model 3= Model 1 + “insulin change”), or “insulin change” and “BMI change” during follow-up (Model 4= Model 1 + “BMI change” + “insulin change”). Model quality was analyzed by evaluating the area under the curve of the receiver operating characteristic curve.

In subanalyses, based on the assumption that BMI and fasting insulin are possible major contributing factors for the incidence of increased IMT and arteriosclerosis, both variables were divided into quartiles and changes from baseline to follow-up were parted in tertiles. Furthermore, relative risk (RR) and adjusted relative risk (ARR) for increased IMT and arteriosclerosis were calculated. ARR was analyzed due to Mantel-Haenszel analysis stratifying for significant covariables. RR analyses were based on the strongest predictors identified due to the logistic regression analysis. For this purpose, fasting insulin, BMI and age were recoded into dichotomous variables defined as hyperinsulinemia (fasting insulin: >15 μU/ml), overweight (BMI: ≥25 kg/m^2^) and older age (>44.5 years).

Level of significance was set at α = 0.05 from two-sided tests. Statistical analyses were performed using GraphPad Prism 6.04 (GraphPad Software, San Diego, CA, USA) and SPSS 22.0 (SPSS Inc., Chicago, IL, USA).

## Results

### Study Population

From *n* = 6,825 included employees, *n* = 3,332 participants already had their second visit in this open-label study. Based on this cohort, *n* = 1,327 participants were excluded because of an incomplete data set, but their baseline characteristics were not different from the cohort with complete data sets (Supplementary Table 1). An additional 366 participants were excluded for the analysis because of cardiovascular impairments at baseline, such as increased IMT, arteriosclerosis or other forms of cardiovascular disease (e.g., stroke, myocardial infarction) (Supplementary Figure 1). Characteristics of the study population (*n* = 1,639) are shown in Table 1.

**Table 1:** Characteristics of study population at baseline and at the first follow-up.

**Anthropometrical and clinical parameter (*n* = 1,639)**	**Baseline (T0)**	**Follow-up (T1)**	**P**
Age (years)	45 ± 5	50 ± 4	<0.001
Sex [m] (%)	49.4	49.4	-
BMI (kg/m^2^)	25.6 ± 4.0	25.9 ± 4.2	<0.001
Systolic blood pressure (mmHg)	126 ± 16	129 ± 16	<0.001
Diastolic blood pressure (mmHg)	82 ± 10	81 ± 10	0.001
HDL-C (mg/dl)	62 ± 16	64 ± 17	<0.001
LDL-C (mg/dl)	126 ± 33	129 ± 34	<0.001
Total cholesterol (mg/dl)	207 ± 36	209 ± 37	0.005
Triglycerides (mg/dl)	89 (61)	87 (59)	0.008
Fasting insulin (μU/ml)	6.7 (5.0)	7.2 (5.0)	<0.001
Fasting blood glucose (mg/dl)	89 ± 11	88 ± 11	<0.001
HOMA-IR	1.44 (1.20)	1.53 (1.14)	0.062
HbA1c (%)	5.37 ± 0.36	5.53 ± 0.35	<0.001
Hypertension (%)	28.0	30.4	0.055
Hypertriglyceridemia (%)	16.4	14.6	0.048
Hypercholesterolemia (%)	55.7	58.4	0.027
Hyperinsulinemia (%)	7.9	9.3	0.086
Insulin resistance (%)	17.3	18.5	0.234
Type 2 diabetes mellitus (%)	1.3	2.3	0.815
Increased IMT (%)	0	14.5	-
Arteriosclerosis (%)	0	19.2	-
Smoking* (%)	12.0	15.2	<0.001

Data are shown as mean ± standard deviation, median (interquartile range), or percentages. *Smoking status includes former smoker, current smoker and ever smoker; definition of cardiometabolic diseases: hypercholesterolemia: ≥200 mg/dl, hypertension: systolic blood pressure: ≥140 mmHg and/or diastolic blood pressure: ≥90 mmHg, hypertriglyceridemia: triglycerides: ≥ 150 mg/dl, insulin resistance: HOMA-IR: ≥2.6, hyperinsulinemia: fasting insulin: >15 μU/ml; type 2 diabetes mellitus: fasting blood glucose: ≥126mg/dl and/or HbA1c ≥6.5%.

*BMI, body-mass-index; HbA1c, hemoglobin A1c; HDL-C, high-density lipoprotein cholesterol; HOMA-IR, homeostasis model assessment of insulin resistance; IMT, intima-media thickness; LDL-C, low-density lipoprotein cholesterol; m, male*.

### Incidence of Increased IMT and Arteriosclerosis

During a mean follow-up time of 5 ± 1 years, 238 participants (15 %) developed increased IMT and 328 (20 %) were diagnosed with atherosclerosis. The association of these endpoints with baseline or follow-up characteristics was analyzed by four different approaches (*Model 1–4*).

#### Increased IMT and Associated Risk Factors

In the initial model (*Model 1*), which focusses only on baseline characteristics, age, smoking, BMI, fasting insulin, and diastolic blood pressure associated with the incidence of increased IMT (Table 2). These associations were not modified after additional adjustment for BMI change during follow-up (*Model 2*). BMI change itself was associated with increased IMT. Additional adjustment for fasting insulin change (instead of BMI change) during follow-up (*Model 3*) destroyed the role of baseline BMI as risk factor but maintained all other baseline risk factors. Fasting insulin change itself associated with increased IMT. The combined adjustment for changes of BMI and fasting insulin during follow-up (*Model 4*) supported the stronger association of fasting insulin compared to BMI. Adjustment for fasting insulin change eliminated the association of BMI change with increased IMT, but not vice versa (Table 2). HOMA-IR did not associate with the incidence of increased intima media thickness in any model.

**Table 2:** Baseline characteristics and changes of BMI and/or fasting insulin during follow-up as risk factors of increased IMT and arteriosclerosis.

	**Model 1**		**Model 2**		**Model 3**		**Model 4**	
**Parameter**	**OR [95% CI]**	***P***	**OR [95% CI]**	***P***	**OR [95% CI]**	***P***	**OR [95% CI]**	***P***
**Increased IMT (*****n*** **=** **238)**								
Age (years)	1.103 (1.071; 1.136)	** <0.001**	1.105 (1.073; 1.138)	** <0.001**	1.107 (1.074; 1.140)	** <0.001**	1.108 (1.076; 1.142)	** <0.001**
Sex (m) (%)	1.250 (0.890; 1.750)	0.196	1.281 (0.902; 1.798)	0.153	1.275 (0.906; 1.795)	0.163	1.292 (0.912; 1.817)	0.143
Smoking (yes)	2.630 (1.630; 4.220)	** <0.001**	2.572 (1.600; 4.130)	** <0.001**	2.640 (1.632; 4.270)	** <0.001**	2.600 (1.601; 4.205)	** <0.001**
BMI (kg/m^2^)	1.045 (1.002; 1.090)	**0.040**	1.048 (1.004; 1.093)	**0.032**	1.024 (0.981; 1.070)	0.278	1.028 (0.986; 1.074)	0.223
Fasting insulin (μU/ml)	1.036 (1.009; 1.064)	**0.008**	1.037 (1.009; 1.066)	**0.010**	1.076 (1.044; 1.110)	** <0.001**	1.073 (1.040; 1.108)	** <0.001**
Fasting blood glucose (mg/dl)	0.994 (0.980; 1.009)	0.430	0.994 (0.980; 1.009)	0.449	0.996 (0.982; 1.011)	0.643	0.996 (0.982; 1.011)	0.642
Systolic blood pressure (mmHg)	1.007 (0.993; 1.022)	0.333	1.007 (0.992; 1.022)	0.346	1.007 (0.992; 1.021)	0.380	1.007 (0.992; 1.021)	0.384
Diastolic blood pressure (mmHg)	1.030 (1.010; 1.050)	**0.009**	1.030 (1.008; 1.051)	**0.009**	1.032 (1.010; 1.053)	**0.005**	1.032 (1.009; 1.053)	**0.005**
HDL-C (mg/dl)	1.005 (0.985; 1.026)	0.602	1.006 (0.986; 1.026)	0.572	1.006 (0.986; 1.026)	0.561	1.006 (0.984; 1.027)	0.544
LDL-C (mg/dl)	1.013 (0.995; 1.031)	0.156	1.013 (0.995; 1.031)	0.157	1.012 (0.996; 1.030)	0.208	1.012 (0.994; 1.030)	0.204
Total cholesterol (mg/dl)	1.006 (0.977; 1.012)	0.552	0.995 (0.977; 1.012)	0.542	0.996 (0.979; 1.014)	0.653	0.996 (0.979; 1.014)	0.656
Triglycerides (mg/dl)	1.002 (0.998; 1.005)	0.295	1.002 (0.998; 1.005)	0.307	1.001 (0.998; 1.004)	0.412	1.001 (0.998; 1.005)	0.410
HbA1c (%)	1.194 (0.740; 1.486)	0.358	1.162 (0.700; 1.462)	0.433	1.294 (0.890; 1.456)	0.136	1.274 (0.850; 1.533)	0.177
HOMA-IR	1.161 (0.711; 1.895)	0.552	1.320 (0.826; 2.109)	0.245	1.155 (0.699; 1.909)	0.574	1.302 (0.807; 2.099)	0.279
BMI change (kg/m^2^)	-	-	1.102 (1.012; 1.200)	**0.026**	-	-	1.063 (0.974; 1.160)	0.168
Insulin change (μU/ml)	-	-		-	1.055 (1.030; 1.082)	** <0.001**	1.052 (1.025; 1.078)	** <0.001**
**Arteriosclerosis (*****n*** **=** **314)**								
Age (years)	1.008 (0.980; 1.036)	0.543	1.010 (0.982; 1.038)	0.493	1.010 (0.982; 1.038)	0.499	1.010 (0.982; 1.039)	0.483
Sex (m) (%)	1.693 (1.245; 2.305)	**0.001**	1.722 (1.259; 2.354)	**0.001**	1.743 (1.273; 2.389)	**0.001**	1.750 (1.282; 2.401)	**0.001**
Smoking (yes)	3.420 (2.200; 5.321)	** <0.001**	3.380 (2.173; 5.256)	** <0.001**	3.540 (2.265; 5.430)	** <0.001**	3.520 (2.254; 5.500)	** <0.001**
BMI (kg/m^2^)	1.052 (1.013; 1.093)	**0.009**	1.055 (1.015; 1.096)	**0.007**	1.031 (0.991; 1.073)	0.128	1.033 (0.992; 1.075)	0.115
Fasting insulin (μU/ml)	1.025 (1.002; 1.049)	**0.037**	1.025 (1.001; 1.050)	**0.049**	1.068 (1.036; 1.101)	** <0.001**	1.066 (1.034; 1.100)	** <0.001**
Fasting blood glucose (mg/dl)	1.002 (0.998; 1.004)	0.994	1.001 (0.997; 1.014)	0.957	1.002 (0.989; 1.016)	0.726	1.002 (0.989; 1.016)	0.722
Systolic blood pressure (mmHg)	1.010 (0.998; 1.023)	0.148	1.010 (0.996; 1.023)	0.157	1.010 (0.996; 1.023)	0.157	1.010 (0.996; 1.023)	0.159
Diastolic blood pressure (mmHg)	1.018 (0.998; 1.039)	0.077	1.018 (0.998; 1.039)	0.078	1.017 (0.996; 1.038)	0.112	1.017 (0.996; 1.038)	0.112
HDL-C (mg/dl)	1.004 (0.985; 1.022)	0.703	1.004 (0.985; 1.022)	0.697	1.005 (0.986; 1.023)	0.629	1.005 (0.986; 1.023)	0.629
LDL-C (mg/dl)	1.005 (0.987; 1.024)	0.604	1.005 (0.988; 1.021)	0.626	1.003 (0.986; 1.020)	0.738	1.003 (0.986; 1.020)	0.742
Total cholesterol (mg/dl)	1.003 (0.987; 1.019)	0.691	1.004 (0.998; 1.020)	0.658	1.005 (0.989; 1.022)	0.535	1.005 (0.989; 1.022)	0.529
Triglycerides (mg/dl)	1.001 (0.997; 1.004)	0.998	1.001 (0.997; 1.004)	0.983	1.001 (0.999; 1.002)	0.869	1.001 (0.997; 1.003)	0.871
HbA1c (%)	2.067 (1.410; 3.010)	** <0.001**	2.119 (1.402; 3.202)	** <0.001**	1.881 (1.238; 2.657)	**0.003**	1.901 (1.249; 2.894)	**0.003**
HOMA-IR	1.100 (0.720; 1.682)	0.659	1.277 (0.797; 2.048)	0.310	1.090 (0.714; 1.663)	0.690	1.267 (0.792; 2.029)	0.324
BMI change (kg/m^2^)	-	-	1.062 (0.984; 1.145)	0.124	-	-	1.020 (0.942; 1.104)	0.624
Insulin change (μU/ml)	-	-	-	-	1.057 (1.030; 1.085)	** <0.001**	1.056 (1.028; 1.084)	** <0.001**

*Logistic regression analyses considering age, sex, smoking, BMI, fasting insulin, systolic and diastolic blood pressure, HDL-C, LDL-C, total cholesterol, triglycerides, fasting blood glucose, HbA1c (Model 1), + ‘BMI change' during follow-up (Model 2) or + ‘fasting insulin change' during follow-up (Model 3), or + both, ‘BMI and insulin change' (Model 4) were performed to determine risk factors for the incidence of increased IMT and arteriosclerosis. In each model, each individual parameter was analyzed with logistic regression for all other parameters. Shown are odds ratios (OR) with 95% confidence interval (95% CI).*

*Bold written P-values represent significance.*

*BMI, body-mass-index; HbA1c, hemoglobin A1c; HDL-C, high-density lipoprotein cholesterol; IMT, intima media thickness; LDL-C, low-density lipoprotein cholesterol; m, male*.

#### Arteriosclerosis and Associated Risk Factors

In the basic model (*Model 1*), sex, smoking, BMI, fasting insulin, and HbA1c associated with the incidence of arteriosclerosis (Table 2). These associations were not altered after additional adjustment for BMI change during follow-up (*Model 2*). BMI change itself was not related to arteriosclerosis. Additional adjustment for fasting insulin change (instead of BMI change) during follow-up (*Model 3*) destroyed the role of baseline BMI as risk factor but maintained all other baseline risk factors. Fasting insulin change itself associated with arteriosclerosis. The combined adjustment for changes of BMI and fasting insulin (*Model 4*) supported the stronger association of fasting insulin compared to BMI within 5 years of follow-up (Table 2). HOMA-IR did not associate with the incidence of arteriosclerosis in any model.

#### Incidence of Increased IMT and Arteriosclerosis in Subgroups Defined by BMI or Fasting Insulin

**Figure 1:**
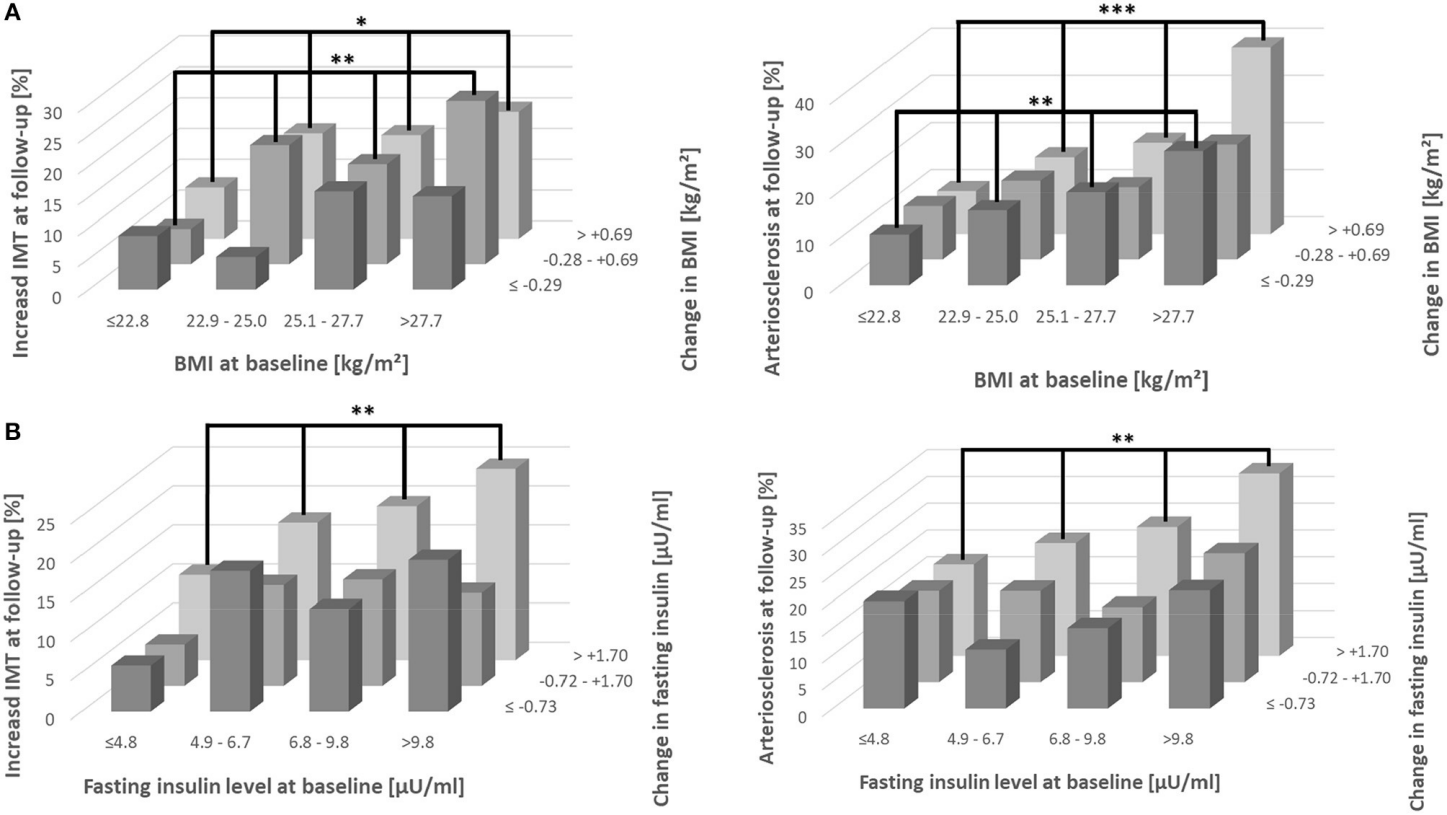
Incidence of increased IMT and arteriosclerosis in subgroups defined by BMI **(A)** or fasting insulin **(B)**. Participants were stratified into subgroups defined by quartiles of baseline BMI or fasting insulin and tertiles of BMI or fasting insulin change during follow-up. Differences in distribution were analyzed using Chi-square test. ***p < 0.001; **p < 0.01; *p < 0.05; IMT, intima media thickness.

**Figure 2:**
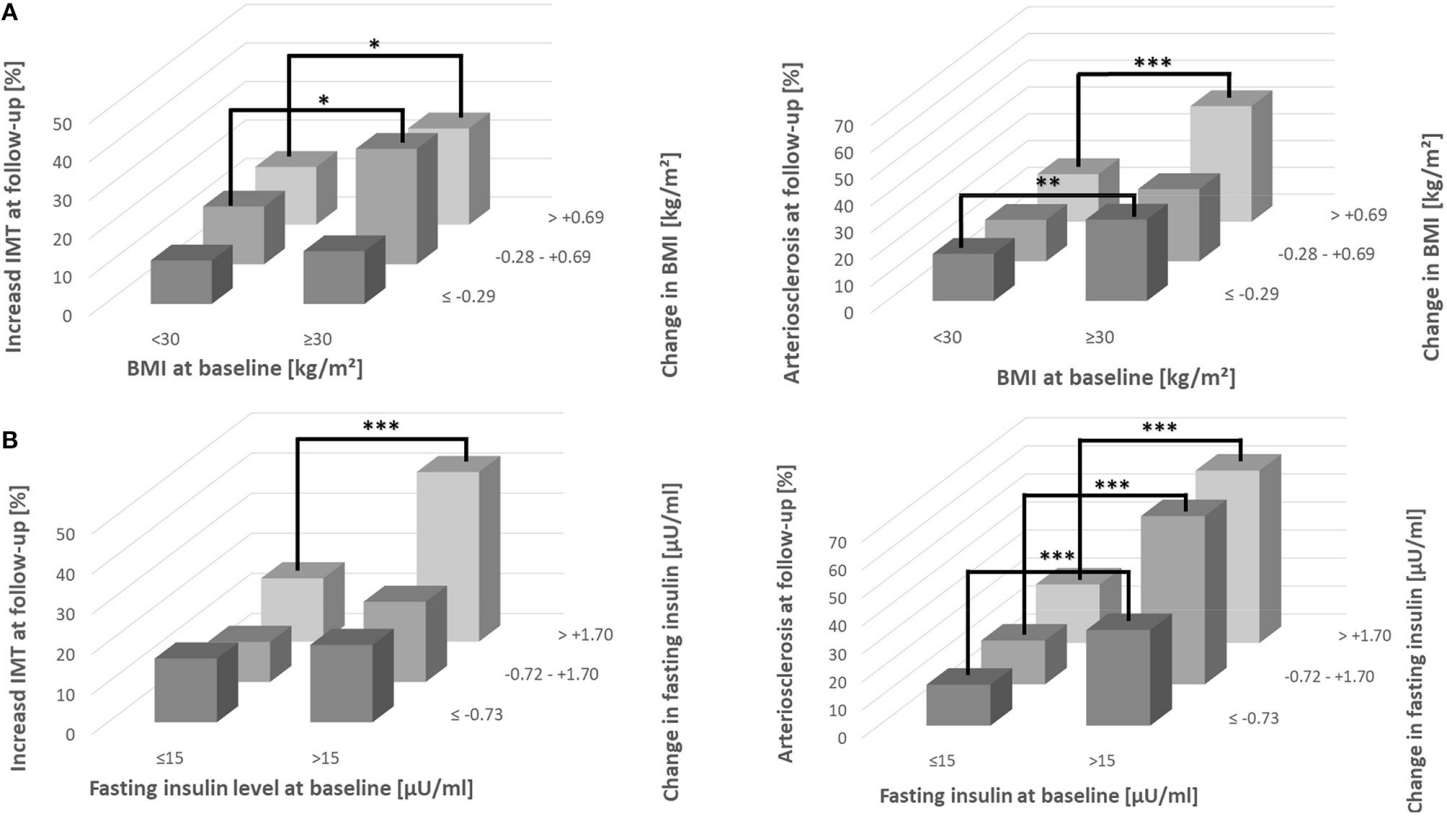
Incidence of increased IMT and arteriosclerosis in subgroups defined by BMI **(A)** or fasting insulin **(B)**. Participants were stratified into subgroups defined by obesity (BMI: ≥30 kg/m^2^) or hyperinsulinemia (fasting insulin: >15 μU/ml) and tertiles of BMI or fasting insulin change during follow-up. Differences in distribution were analyzed using Chi-square test. ***p < 0.001; **p < 0.01; *p < 0.05; IMT, intima media thickness.

When participants were stratified into subgroups defined by quartiles of baseline BMI and tertiles of BMI change during follow-up the subgroup with lowest baseline and follow-up values exhibited the lowest risk of increased IMT or arteriosclerosis (Figure 1A). Risks increased with higher baseline and follow-up values and highest risks were observed in the group with highest baseline BMI values. Similar results were seen after stratification of baseline and follow-up values of fasting insulin. The highest risk of both cardiovascular endpoints was found for the subgroup of highest baseline of fasting insulin combined with greatest change during follow-up (Figure 1B). The data confirm that both, baseline values and changes during follow-up contribute to the risk of cardiovascular endpoints. After recategorization of BMI and fasting insulin baseline quartiles into obesity (≥30 kg/m^2^) (Figure 2A) and hyperinsulinemia (fasting insulin: >15 μU/ml) (Figure 2B), aforementioned associations were even stronger, especially for insulin. An increase in insulin over time and being hyperinsulinemic enhanced the risk of developing increased IMT and arteriosclerosis.

#### Relative and Adjusted Relative Risk of Major Risk Factors for Increased IMT and Arteriosclerosis

**Figure 3:**
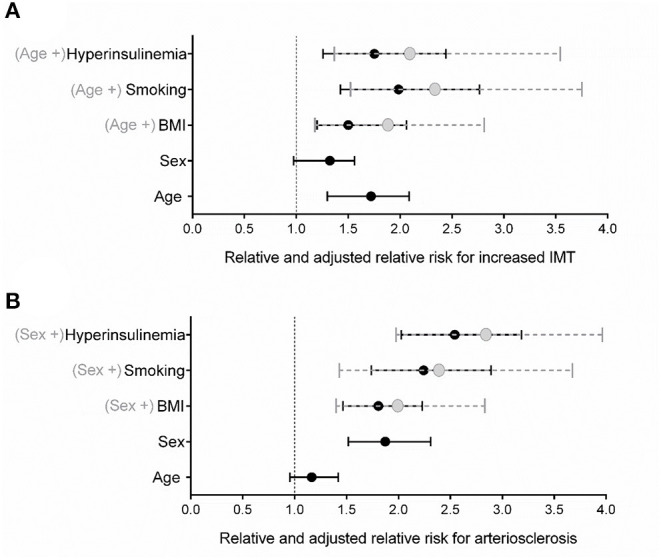
Relative and adjusted relative risk of major risk factors for **(A)** increased IMT and **(B)** arteriosclerosis. Relative risk [mean and 95% CI (in black)] and adjusted relative risk [mean and 95% CI (in gray)] were determined by using Mantel-Haenszel analyses. Prior analysis, parametrical variables were redefined into dichotomous variables including age (> or ≤ 44.5 years), overweight (≥25 kg/m^2^ BMI), and hyperinsulinemia (>15 μU/ml fasting insulin).

After recoding all parametrical characterized parameters into nominal variables, based on their individual median (fasting insulin recoding based on the definition for hyperinsulinemia), all identified risk factors remained significant for predicting increased IMT {RR: mean [95% CI] (BMI: 1.5 [1.2, 2.1]; age: 1.7 [1.3, 2.1]; smoking: 2.0 [1.4, 2.8]; hyperinsulinemia: 1.8 [1.3, 2.4])} or arteriosclerosis {RR: (BMI: 1.8 [1.5, 2.2]; sex: 1.9 [1.5, 2.3]; smoking: 2.2 [1.7, 2.9]; hyperinsulinemia: 2.5 [2.0, 3.2])} as shown before in the logistic regression analyses. A final comparison of BMI and fasting insulin concentrations as cardiovascular risk factor was performed by calculating the adjusted relative risk. The addition of fasting insulin to the risk factors age {for increased IMT (ARR: 2.2 [1.4, 3.6])} or sex {for arteriosclerosis (ARR: 2.8 [1.9, 4.0])} yielded higher relative risks than the addition of BMI with regard to increased IMT (ARR: 1.8 [1.2, 2.8]) (Figure 3A) or arteriosclerosis (ARR: 2.0 [1.4, 2.8]) (Figure 3B). Smoking provided a slightly higher relative and adjusted relative risk for increased IMT (ARR: 2.3 [1.5, 3.7]) than fasting insulin (Figure 3A) and was not stronger associated than fasting insulin as risk factor for arteriosclerosis (ARR: 2.3 [1.3, 3.6]) (Figure 3B). Interestingly, including the parameter smoking did not lead to a higher risk for increased IMT or arteriosclerosis than conferred by high fasting insulin levels.

Further subanalyses indicated that participants with prediabetes (HbA1c: 5.7-6.4 %) and patients with type 2 diabetes (HbA1c: ≥6.5 %) had higher frequencies of increased IMT and arteriosclerosis.

## Discussion

In the present longitudinal study middle-aged employees were followed up in average for 5 years as part of an occupational health care program. As expected, there was incidence or progression of atherogenic lesions, defined by increased IMT and atherosclerosis. Multivariable logistic regression models confirmed common risk factors at baseline such as age, sex, smoking, as well as BMI. We identified fasting insulin blood levels at baseline as a further strong predictor of atherogenic progression. Moreover, comprehensive modeling revealed that in addition insulin changes over time have a significant association with the incidence of increased IMT and arteriosclerosis. Fasting insulin is controversially discussed as a reliable marker for predicting cardiovascular events as not all long-term follow-up studies identified fasting insulin levels as a critical factor for developing CVD (40). Differences between the studies may account for the inconsistency. Welin et al. (follow-up for 8 years) included older aged men (*n* = 595) with high normal or elevated fasting insulin levels (27). Hargreaves et al. (follow-up for 12 years) considered only data of 83 persons in their final logistic regression models of whom 11 developed coronary heart disease (32). Liu et al. did not find associations of fasting insulin concentrations with incident electrocardiogram abnormalities in Pima Indians (*n* = 47 cases in 994 study participants) (26). In smaller cohorts, the authors did not find associations of fasting insulin levels with the progression of carotid intima media thickness (72 adolescent study participants for 2 years) or in 374 young adults after 13 years, as well as in 84 postmenopausal women after 5 years of follow-up, respectively (28–30). The pronounced obesity-related hyperinsulinemia and insulin resistance in Pima Indians may have obscured the association of fasting insulin with markers of CVD risk (41). Another aspect is that we find the increase of fasting insulin levels during follow-up as particularly strong risk factors for endothelial lesions, and this parameter has not been analyzed in these studies.

There are also confirmative studies to the present findings. Strong associations of fasting insulin with a 5-year progression of carotid atherosclerosis (i.e., IMT, plaque presence) have been shown for middle-aged European and Asian persons (31). Furthermore, high-dosages of exogenic induced insulin associated with an increasing CVD risk in older hospitalized patients with type 2 diabetes in an observational study (22). Moreover, patients with atrial fibrillation and insulin-requiring diabetes had an increased thromboembolic risk (stroke/systemic embolism) at 1 year compared to those patients with diabetes but without insulin therapy (23). There are also cross-sectional studies demonstrating significant correlations of fasting insulin levels with different entities of early arteriosclerosis such as carotid artery stiffness in 83 middle-aged patients with hypertension, or increased carotid IMT in 100 healthy middle-aged persons as well as coronary calcification in 443 middle-aged men and women (18, 20, 21).

High fasting insulin levels and hyperinsulinemia, respectively, may promote atherogenesis by inducing endothelial dysfunction (by reducing the activation of endothelial NO synthase) (14), pro-inflammatory activity of macrophages (15), suppression of autophagy and an increase in protein synthesis (e.g., by upregulating the mechanistic target of rapamycin complex 1) (16), as well as a compromised cytoprotective response to oxidative and other chemical stress (by suppressing the nuclear factor Nrf2) (17).

In the present study BMI also significantly contributed to the risk of developing increased IMT and arteriosclerosis. These results are in line with the current literature as BMI is often used in predicting models as a prognostic factor for CVD progression and the incidence of CVE's (42). Besides age, sex, and smoking (1), obesity has been discussed as a major factor for the development of atherosclerosis (43) and hyperinsulinemia and/or insulin resistance are seen as accompanied consequences of excessive weight gain. However, based on the present results, especially when considering changes of fasting insulin and BMI over time, higher fasting insulin levels exhibit a stronger association with increased IMT and arteriosclerosis than BMI. Adjusting for changes of fasting insulin blood levels during follow-up eliminated BMI as risk factor but not vice versa. These findings are similar to a meta-analysis of hyperinsulinemia vs. risk of cardiovascular mortality in men and women with type 2 diabetes which also reported an association independent of BMI (44). Based on the current findings, the broadly accepted causal model between obesity, the resulting hyperinsulinemia, and atherogenic progression is obsolete. Moreover, fasting insulin levels should be also evaluated at least in a similar fashion in comparison to BMI from a clinical perspective for preventing cardiovascular diseases. Furthermore, the current results support the potential role of high insulin levels for atherogenic progression and that maybe obesity is rather a consequence than the actual risk factor for developing hyperinsulinemia and in the further course arteriosclerosis (8).

In the present study, multivariable logistic regression modeling identified smoking as an additional independent risk factor for developing increased IMT and arteriosclerosis. Interestingly, hyperinsulinemia was stronger associated with arteriosclerosis than smoking. Further adjusted relative risk analyses found that relative risks conferred by smoking and hyperinsulinemia were not additive. This finding indicates that smoking and hyperinsulinemia target the same atherogenic mechanism. Hyperinsulinemic persons tend to have an insulinotropic effect by smoking (45) and chronic smokers with (46) or without diabetes (47) have a significantly reduced insulin sensitivity which in turn led to hyperinsulinemia.

Furthermore, HOMA-IR did not associate with the incidence of increased IMT or arteriosclerosis in the present study. Studies supporting insulin resistance as a risk factor for atherogenic progression differ in part significantly to the present study, especially in case of population size (n <100) or age (≥ 70 years) (33–35), study design (e.g., cross-sectional design) (37), follow-up period (1 year) (48), and fasting insulin levels were not treated as a confounder in the statistical analyses. Interestingly, Hanley et al., the only study which equally investigated HOMA-IR and fasting insulin with a predictive approach (follow-up after 8 years), found that, both, HOMA-IR and fasting insulin were comparable predictors (with similar OR's in comparison to the present study) for the incidence of cardiovascular events in middle-aged persons of the San Antonio Heart Study (49). Possible reasons for the difference to the present study might be ethnical (> 50% Hispanic population) or behavioral (e.g., > 25% active smoker) as well as the primary endpoint of the study (incidence of precursors of CVD vs. incident cardiovascular events). A recently published observational and cross-sectional study with a large cohort (*n* = 5,764) indicates that insulin resistance not consistently associates with the presence of coronary artery disease and that hyperglycemic conditions can have a significant impact in this context (36). The variable findings with regard to HOMA-IR may be due to the weak correlation with direct measures of insulin sensitivity (e.g., euglycaemic–hyperinsulinaemic clamp) (38).

In the present study, fasting blood glucose did also not correlate with the incidence of increased IMT or arteriosclerosis. Other works demonstrated similar results that postprandial glucose levels rather than fasting blood glucose associate with atherogenic progression (50). This interrelation supports the assumption that higher fasting insulin levels or a hyperinsulinemic status rather than elevated fasting blood glucose levels mediate the development of arteriosclerosis (51). Furthermore, fasting blood glucose, triglycerides, HDL cholesterol, as well as diastolic blood pressure did not deteriorate despite the overall BMI increase of 0.3 kg/m^2^ (≈ 1 kg). However, subanalyses revealed that participants experiencing an increase in body weight above 1 kg had expected worsening of triglycerides, fasting blood glucose, HDL cholesterol, and diastolic blood pressure levels. A possible explanation for this data course is that a small increase in BMI of ≤ 0.3 kg/m^2^ over 5 years in a younger working cohort does not inevitably lead to a deterioration of clinical parameters (Supplementary Table 2).

There are strengths and limitations of the present study which should be considered. Although the results are based on a large number of people, it must be taken into account that these are all middle-aged persons. Focusing on this particular cohort might lead to a selection bias as persons in this age tend to have less prevalent endothelial lesions. However, the longer observation period revealed a substantial rise in the number of persons with increased IMT and arteriosclerosis. Furthermore, the results of the present study describe associations and cannot prove causal relationships. Moreover, it needs to be considered that information about medication was not recorded (e.g., antidiabetic, antihyperlipidemic, antihypertensive drugs). Besides medication, there is also missing information regarding ethnic background as well as a detailed recording of comorbidities (e.g., polycystic ovary syndrome, chronic viral hepatitis, chronic renal failure, fatty liver disease, adrenal and pituitary diseases, infectious and oncological diseases).

Strengths of the present work comprise the observation period of 5 years and the large number of persons followed up.

Taken together, fasting insulin levels should be considered in the assessment and prognosis of atherosclerotic progression in addition to the common risk factors age, sex, smoking, and BMI. From a clinical perspective, HOMA-IR appears to be of inferior relevance for predicting precursors of cardiovascular disease. Furthermore, more attention should be paid to the prevention or treatment of hyperinsulinemia because of the associated health risks. One possibility would be the recommendation of a lifestyle favoring low insulin levels as part of general prevention guidelines.

## Data Availability Statement

The datasets presented in this article are not readily available because of legal restrictions. Requests to access the datasets should be directed to martin.roehling@vkkd-kliniken.de.

## Ethics Statement

The studies involving human participants were reviewed and approved by Ärztekammer Nordrhein (no. 2011340). The patients/participants provided their written informed consent to participate in this study.

## Author Contributions

KK, MS, and SM are responsible for the conception and design of the study. MS collected data. KK, MR, HK, TM, and SM analyzed and interpreted data. MR drafted the manuscript. KK, TM, MS, HK, and SM approved the final version of the manuscript. KK and SM are the guarantors of this work and, as such, had full access to all the data in the study and take responsibility for the integrity of the data and the accuracy of the data analysis. All authors contributed to the article and approved the submitted version.

## Funding

This study was funded by Boehringer Ingelheim International GmbH and by Gesellschaft von Freunden und Förderern der Heinrich-Heine-Universität Düsseldorf e.V.

## Conflict of Interest

KK and SM received research support from Boehringer Ingelheim International GmbH & Co. KG. MS is an employee of Boehringer Ingelheim Pharma GmbH & Co. KG. The funders had no role in study design, data collection, data analysis, data interpretation, or writing of the manuscript. The remaining authors declare that the research was conducted in the absence of any commercial or financial relationships that could be construed as a potential conflict of interest.

## Publisher's Note

All claims expressed in this article are solely those of the authors and do not necessarily represent those of their affiliated organizations, or those of the publisher, the editors and the reviewers. Any product that may be evaluated in this article, or claim that may be made by its manufacturer, is not guaranteed or endorsed by the publisher.
