# Supplementary material for: The Epidemiological Boehringer Ingelheim Employee Study (Part 3): Association of Elevated Fasting Insulin Levels but Not HOMA-IR With Increased Intima Media Thickness and Arteriosclerosis in Middle-Aged Persons
Source: Front Cardiovasc Med. 2021 Oct 22;8:752789. doi: 10.3389/fcvm.2021.752789 (PMC8569239; doi:10.3389/fcvm.2021.752789)
Supplement: Supplementary file 1 [file Data_Sheet_1.docx]

**Supplementary fig. 1. Flowchart**

Participants attended **first study visit** (n=6825)

## First visit

**Excluded:**

♦ Second visit not yet happened (n=3493)

Participants attended **second study visit** (n=3332)

## Follow-Up

**Excluded:**

♦ Missing data (n=1327)

## Data availability

**Complete set of data** for all variables (n=2005)

**Excluded:**

♦ Already existent cardiovascular impairments (n=366)

(1) Increased IMT: n=165

(2) Arteriosclerosis: n=182

(3) Cardiovascular disease: n=19 (e.g. stroke, myocardial infarction)

**Incidence:**

(1) Increased IMT (n=238)

(2) Arteriosclerosis (n=314)

## Outcome

## Analysis

**Eligible data for incidence analysis** (n=1639)
