# Supplementary material for: The Epidemiological Boehringer Ingelheim Employee Study (Part 3): Association of Elevated Fasting Insulin Levels but Not HOMA-IR With Increased Intima Media Thickness and Arteriosclerosis in Middle-Aged Persons
Source: Front Cardiovasc Med. 2021 Oct 22;8:752789. doi: 10.3389/fcvm.2021.752789 (PMC8569239; doi:10.3389/fcvm.2021.752789)
Supplement: Supplementary file 2 [file Data_Sheet_2.docx]

**Supplementary table 1. Characteristics of included and excluded participants with an incomplete set of data at baseline.**

| Anthropometrical and clinical parameter | Study population  (n=1639) | Incomplete set of data  (n=1327) |
| --- | --- | --- |
| Age (years) | 45±5 | 46±5 |
| Sex [m] (%) | 49.4 | 52.8 |
| BMI (kg/m²) | 25.6±4.0 | 25.9±4.2 |
| Systolic blood pressure (mmHg) | 126±16 | 125±16 |
| Diastolic blood pressure (mmHg) | 82±10 | 81±10 |
| HDL-C (mg/dl) | 62±16 | 61±16 |
| LDL-C (mg/dl) | 126±33 | 129±32 |
| Total cholesterol (mg/dl) | 207±36 | 208±35 |
| Triglycerides (mg/dl) | 89 (61) | 96 (69) |
| Fasting insulin (µU/ml) | 6.7 (5.0) | 7.1 (5.2) |
| Fasting blood glucose (mg/dl) | 89±11 | 90±15 |
| HOMA-IR | 1.44 (1.20) | 1.56 (1.21) |
| HbA1c (%) | 5.37±0.36 | 5.38±0.41 |
| Hypertension (%) | 28.0 | 27.2 |
| Hypertriglyceridemia (%) | 16.4 | 20.6 |
| Hypercholesterolemia (%) | 55.7 | 57.8 |
| Hyperinsulinemia (%) | 7.9 | 11.4 |
| Insulin resistance (%) | 17.3 | 19.9 |
| Type 2 diabetes mellitus (%) | 1.3 | 2.2 |
| Increased IMT (%) | 0 | 10.5 |
| Arteriosclerosis (%) | 0 | 9.3 |
| Smoking (%)* | 12.0 | 13.8 |

Data are shown as mean±standard deviation, median (interquartile range), or percentages. *Smoking status includes former smoker, current smoker and ever smoker; definition of cardiometabolic diseases: hypercholesterolemia: ≥200 mg/dl, hypertension: systolic blood pressure: ≥140 mmHg and/or diastolic blood pressure: ≥90 mmHg, hypertriglyceridemia: triglycerides: ≥150 mg/dl, insulin resistance: HOMA-IR: ≥2.6, hyperinsulinemia: fasting insulin: >15 𝜇U/ml and type 2 diabetes mellitus: fasting blood glucose: ≥126mg/dl and/or HbA1c ≥6.5%; BMI, body-mass-index; HbA1c, hemoglobin A1c; HDL-C, high-density lipoprotein cholesterol; HOMA-IR, homeostasis model assessment of insulin resistance; IMT, intima-media thickness; LDL-C, low-density lipoprotein cholesterol; m, male
