# Supplementary material for: The Epidemiological Boehringer Ingelheim Employee Study (Part 3): Association of Elevated Fasting Insulin Levels but Not HOMA-IR With Increased Intima Media Thickness and Arteriosclerosis in Middle-Aged Persons
Source: Front Cardiovasc Med. 2021 Oct 22;8:752789. doi: 10.3389/fcvm.2021.752789 (PMC8569239; doi:10.3389/fcvm.2021.752789)
Supplement: Supplementary file 3 [file Data_Sheet_3.docx]

**Supplementary table 2. Body weight change associated influences on diastolic blood pressure, HDL cholesterol, triglycerides, and fasting blood glucose.**

| Parameter  (n=1639) | >0 kg weight loss  (n=729) | 0-1 kg weight change  (n=212) | 1-5 kg weight gain  (n=502) | >5 kg weight gain  (n=196) |
| --- | --- | --- | --- | --- |
| Diastolic blood pressure (mmHg) | -3±9 | 0±9 | 1±9 | 3±10 |
| HDL cholesterol  (mg/dl) | 4±10 | 2±9 | -1±10 | -3±9 |
| Triglycerides  (mg/dl) | -12±53 | -6±54 | 5±45 | 9±62 |
| Fasting blood glucose (mg/dl) | -4±11 | -1±9 | 2±10 | 4±9 |

Data are shown as mean ± SD. HDL cholesterol, high-density lipoprotein cholesterol. Change in body weight from baseline to the first follow-up grouped in four parts differencing between weight loss (< 0 kg), being weight stable (0-1 kg weight gain), small weight gain (1-5 kg), and large weight gain (> 5 kg).
